# The differential *in vivo* contribution of spinal α_2A_- and α_2C_-adrenoceptors in tonic and acute evoked nociception in the rat

**DOI:** 10.3389/fphar.2022.1023611

**Published:** 2022-11-23

**Authors:** Gustavo López-Córdoba, Guadalupe Martínez-Lorenzana, Jair Lozano-Cuenca, Miguel Condés-Lara, Abimael González-Hernández

**Affiliations:** ^1^ Departamento de Neurobiología Del Desarrollo y Neurofisiología, Instituto de Neurobiología, Universidad Nacional Autónoma de México, Querétaro, Mexico; ^2^ Departamento de Biología Celular, Secretaría de Salud, Instituto Nacional de Perinatología, Mexico City, Mexico

**Keywords:** adrenoceptor, analgesia, clonidine, electrophysiology, pain, behavior

## Abstract

Spinal α_2_-adrenoceptor induces analgesia by neuronal inhibition of primary afferent fibers. This family receptor coupled to G_*i/o*_ proteins can be subdivided into three functional subtypes: α_2A_, α_2B,_ and α_2C_-adrenoceptors, and current evidence on spinal analgesia supports the relevance of α_2A_ and seems to exclude the role of α_2B_, but the functional contribution of α_2C_-adrenoceptors remains elusive. The present study was designed to pharmacologically dissect the contribution of spinal α_2_-adrenoceptor subtypes modulating tonic or acute peripheral nociception. Using male Wistar rats, we analyzed the effect of spinal clonidine (a non-selective α_2A/_α_2B/_α_2C_-adrenoceptor agonist) and/or selective subtype α_2_-adrenoceptor antagonists on: 1) tonic nociception induced by subcutaneous formalin (flinching behavior) or 2) acute nociception induced by peripheral electrical stimulus in *in vivo* extracellular recordings of spinal dorsal horn second-order wide dynamic range (WDR) neurons. Clonidine inhibited the nocifensive behavior induced by formalin, an effect blocked by BRL 44408 (α_2A_-adrenoceptor antagonist) but not by imiloxan (α_2B_-adrenoceptor antagonist) or JP 1302 (α_2C_-adrenoceptor antagonist). Similarly, spinal BRL 44408 reversed the clonidine-induced inhibition of nociceptive WDR activity. Interestingly, spinal JP 1302 *per se* produced behavioral antinociception (an effect blocked by bicuculline, a preferent GABA_A_ channel blocker), but no correlation was found with the electrophysiological experiments. These data imply that, at the spinal level, 1) presynaptic α_2A_-adrenoceptor activation produces antinociception during acute or tonic nociceptive stimuli; and 2) under tonic nociceptive (inflammatory) input, spinal α_2C_-adrenoceptors are pronociceptive, probably by the inactivation of GABAergic transmission. This result supports a differential role of α_2A_ and α_2C_-adrenoceptors modulating nociception.

## 1 Introduction

At the spinal dorsal horn level, α_2_-adrenoceptor activation has been related to the inhibition of neural transmission and analgesic actions (Pertovaara, 2006; Bahari and Meftahi, 2019). From a mechanistic perspective, activation of this receptor which is canonically coupled to G_*i/o*_ proteins, inhibits adenylyl cyclase, inactivates Ca^2+^ channels, and enhances inwardly rectifying K^+^ channel activity, leading to neuronal hyperpolarization with a diminution of neural transmission (Pan et al., 2008). *In vivo* electrophysiological recordings of second-order wide dynamic range (WDR) cells in the spinal dorsal horn have shown that spinal clonidine (α_2_-adrenoceptor agonist) selectively inhibits the neuronal activity of nociceptive primary afferent fibers (Sullivan et al., 1987). As reviewed by Pertovaara (2013), agonists to this receptor consistently produce antinociception in behavioral and electrophysiological experiments. Indeed, intrathecal clonidine has been successfully used in humans as a potent analgesic (Eisenach et al., 1996; Yaksh et al., 2017; Schwartz et al., 2022).

However, we must keep in mind that α_2_-adrenoceptors have been divided into three functional subtypes, namely α_2A_, α_2B_, and α_2C_ (Bylund et al., 1994), and researchers have attempted to dissect how these receptor subtypes contribute to spinal pain transmission. Briefly, molecular assays suggest that the three receptor subtypes are expressed in the spinal cord and dorsal root ganglion cells (Aoki et al., 1994; Stone et al., 1998; Nicholson et al., 2005), and current functional evidence supports the notion that α_2A_-adrenoceptor activation plays a key role in spinal antinociception, whereas α_2B_-adrenoceptors seem not to contribute to spinal antinociception (for refs., see Pertovaara, 2006; Pertovaara, 2013). In contrast, in the case of α_2C_-adrenoceptors, although Fairbanks et al. (2002) suggest that spinal activation of this subtype receptor plays an antinociceptive role, the evidence offered by Malmberg et al. (2001) refuses these data showing that this receptor does not play any role in nociception. In both cases, the experiments were performed using transgenic mice and non-selective ligands. Hence, the function of this subtype receptor remains obscure.

Most importantly, although current literature supports the notion that the main effect of α_2_-adrenoceptors is inhibition of nociceptive transmission, some *in vitro/in vivo* experiments suggest that noradrenergic transmission at the spinal cord level could also be pronociceptive (Hantman and Perl, 2005; Chen et al., 2008; Gassner et al., 2009; Hickey et al., 2014; Kucharczyk et al., 2022). For example, using spinal cord slices and electrophysiological recordings (patch-clamp) at lamina II/III, it has been shown that some GABAergic neurons can be hyperpolarized by noradrenaline via α_2_-adrenoceptor activation (Gassner et al., 2009); in this study, the subtype receptor was not analyzed. Furthermore, also in spinal cord slices, but using immunohistochemistry, Chen et al. (2008) showed that α_2C_-adrenoceptor activation inhibits the veratridine-induced opioid release. These *in vitro* data may imply that α_2C_-adrenoceptors may play a pronociceptive role. In any case, the functional role of α_2C_-adrenoceptors modulating spinal nociception has been hampered (contradictory) in part by the lack of selective ligands used.

On this basis, the present study aimed to functionally dissect α_2A/2B/2C_-adrenoceptors in spinal nociception. Here, we analyzed the effect of spinal clonidine (a non-selective α_2A/_α_2B/_α_2C_-adrenoceptor agonist) and/or selective subtype α_2A/_α_2B/_α_2C_ -adrenoceptor antagonists (see Table 1) on: 1) tonic nociception (flinching behavior) induced by subcutaneous formalin or 2) acute nociception induced by peripheral electrical stimulus in *in vivo* extracellular recordings of spinal dorsal horn second-order wide dynamic range (WDR) neurons. The data showed that the presynaptic α_2A_-adrenoceptor produces robust antinociception during acute or tonic nociceptive stimuli. In contrast, under a tonic nociceptive stimulus, α_2C_-adrenoceptors seem to induce nociception by inactivating GABAergic transmission.

**TABLE 1:** Binding affinity constants for the α_2A_-, α_2B_-, and α_2A_-adrenoceptors of clonidine and the antagonists used in the present study. The nanomoles (and their equivalents in μg) spinally administered in the current experiments are also shown.

	Affinity values (p*K*_i_ or p*K*_D_) for α_2_-adrenoceptor subtypes			Nanomoles (nmol) and their equivalents in μg used in the present study
	α_2A_	α_2B_	α_2C_	
Clonidine^a^	7.2	7.2	6.9	0.1 nmol (0.02 μg)
				1.0 nmol (0.2 μg)
				10.0 nmol (2.0 μg)
BRL 44408^b^	7.2	5.4	6.2	0.1 nmol (0.22 μg)
				1.0 nmol (2.2 μg)
Imiloxan^b^	5.9	6.5	6.3	10 nmol (2.44 μg)
JP-1302^b^	5.3	5.1	6.9	1 nmol (0.37 μg)
				10 nmol (3.7 μg)

All data are given as p*K*_i_.

^a^ (Jasper et al., 1998) or p*K*_D_.

^b^ (Proudman et al., 2022) values at human receptors.

## 2 Material and methods

### 2.1 Experimental animals and ethical standards

**FIGURE 1:**
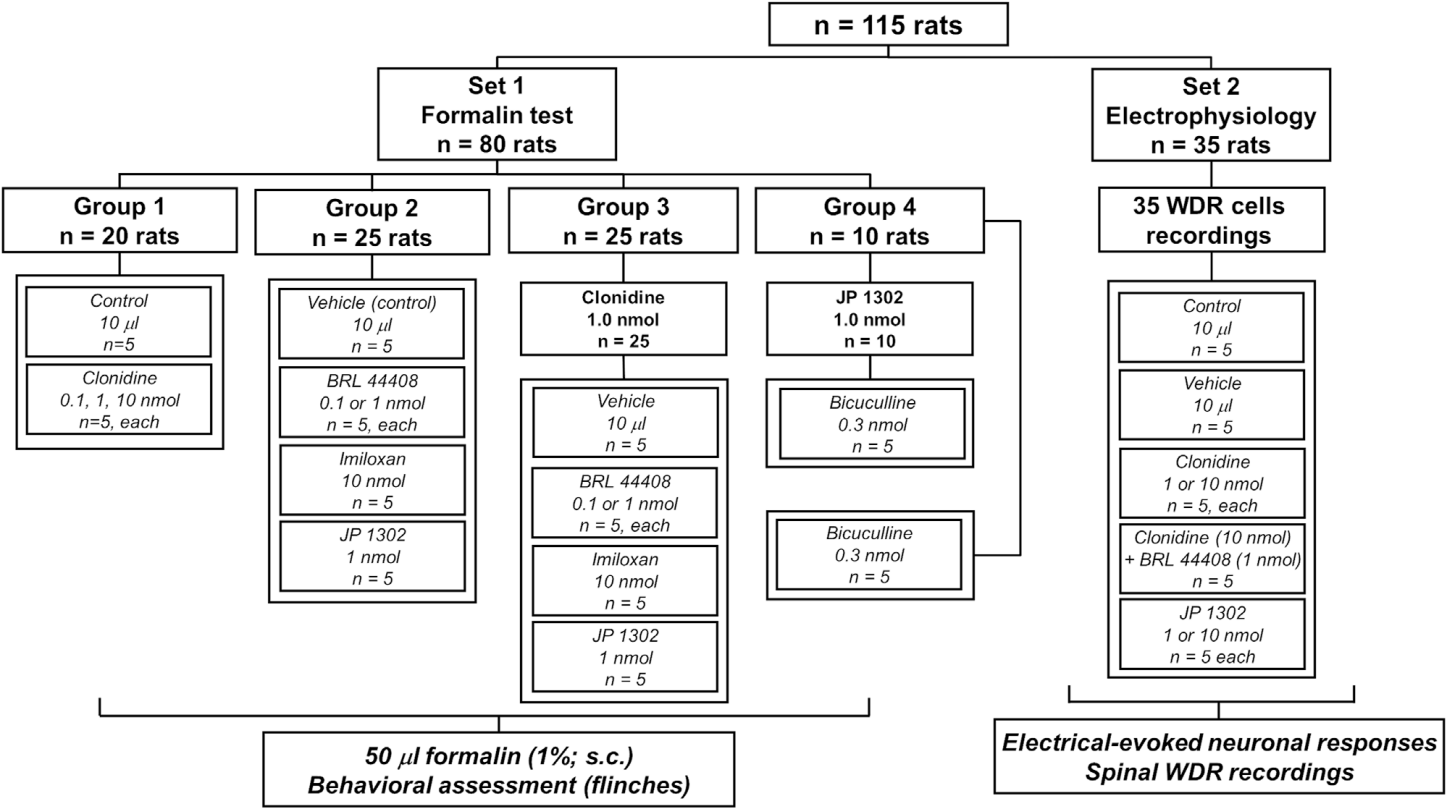
Experimental protocols showing the number of animals used in the present study. The animals were divided into two main sets to perform behavioral or *in vivo* electrophysiological recordings.

A total of 115 male Wistar rats (295 ± 15 g) from the Neurobiology Institute Animal House were used in the present experiments. Rats were divided into two main sets (80 rats for behavioral tests and 35 rats for electrophysiological recordings; see Figure 1). The rodents were housed in the satellite bioterium of our laboratory on a 12:12 h light and dark cycle (lights on at 07:00 h) at constant temperature (22°C ± 2°C) and humidity (50%) with food and water *ad libitum*. All experimental procedures were performed during the light phase of the cycle (10:00–19:00 h). Furthermore, all animal protocols in this investigation were approved by our Institutional Ethics Committee, following the Guide for the Care and Use of Laboratory Animals in the United States (NIH publication 86-23), IASP ethical guidelines (Zimmermann, 1983), and ARRIVE guidelines for reporting experiments involving animals (McGrath et al., 2010). At the end of the experiments, the animals were halted in a CO_2_ chamber (formalin test) or by an overdose of pentobarbital (electrophysiological experiments).

### 2.2 General methods

#### 2.2.1 Surgical procedures for behavioral experiments (intrathecal surgery)

Chronic catheterization of the intrathecal (i.t.) subarachnoid space was performed as described by Yaksh and Rudy (1976). The rats were anesthetized with a ketamine-xylazine (75–10 mg/kg, i.p.) and placed in a stereotaxic frame (Kopf Instruments, United States) with the dorsal part of the head and neck previously shaved. The atlanto-occipital membrane was exposed and pierced, and a polyethylene catheter (PE-10, 9.0 cm length) was inserted intrathecally and advanced caudally to the level of the thoracolumbar junction. The wound was then sutured, and the animals were housed in individual cages to recover from surgery for 5 days before the formalin test.

#### 2.2.2 Formalin-induced tonic nociception and study design

The tonic nociception experiments using the 1% formalin test (Dubuisson and Dennis, 1977; Wheeler-Aceto and Cowan, 1991) were performed by the same tester, blinded to the pharmacological treatment. Rats were placed in open Plexiglass^®^ observation chambers for 30 min for three consecutive days to allow them to become familiar with their surroundings. On the third day, and after 30 min in the Plexiglass^®^ chamber, they were removed for formalin administration. Fifty microliters of diluted formalin (1%) were subcutaneously (s.c.) injected into the dorsal surface of the right hind paw with a 30-gauge needle. The animals were then returned to the chambers, and nocifensive behavior was observed immediately after the formalin injection. Nocifensive behavior was quantified as the number of flinches of the injected paws during 1 min periods every 5 min for up to 1 h after injection. Flinching was characterized as a rapid and brief withdrawal or as a flexing of the injected paw. As previously reported, formalin-induced flinching behavior was biphasic. The initial nociceptive phase (0–10 min) was followed by a prolonged, persistent response (15–60 min). For i.t., administration, the α_2A/2B/2C_-adrenoceptor agonist (clonidine) or selective antagonist (BRL 44408 to α_2A_-, imiloxan to α_2B_-, or JP 1302 to α_2C_-adrenoceptors) were given in a volume of 10 μl using a Hamilton^®^ syringe.

The first group (*n* = 20 rats) was subdivided into four subgroups and received an i.t., injection of vehicle (saline solution, 0.9% NaCl; *n* = 5) or clonidine (0.1, 1 or 10 nmol; *n* = 5 rats each) 10 min before formalin injection. A second group (*n* = 25 rats) was subdivided into five subgroups to measure the *per se* effect of vehicle (*n* = 5) or the antagonists, BRL 44408 (0.1 or 1 nmol; *n* = 5 rats each), imiloxan (10 nmol, *n* = 5) or JP 1302 (1 nmol; *n* = 5 rats each) given 20 min before formalin injection.

To determine whether clonidine-induced intrathecal antinociception was mediated by either α_2A_-, α_2B_-, or α_2C_-adrenoceptors, a third group was used (*n* = 25 rats). In this case, the effect of pretreatment (10 min before 1 nmol clonidine) with vehicle (*n* = 5), BRL 44408 (0.1 or 1 nmol; *n* = 5 rats each), imiloxan (10 nmol; *n* = 5) or JP 1302 (1 nmol; *n* = 5) on the formalin-induced flinches was assessed.

Since JP 1302 alone inhibited the formalin-induced nociception, the involvement of GABAergic mechanisms was suspected (see discussion section). To test this hypothesis, 0.3 nmol bicuculline was given 10 min before JP 1302 (*n* = 5 rats; 1 nmol). Also, the *per se* effect of bicuculline on formalin flinches was assessed (*n* = 5 rats).

Doses and drug administration schedules for clonidine, BRL 44408, and imiloxan were selected based on previous reports (O’Neill and Haigler, 1985; Young et al., 1989; Omote et al., 1991; Uhlén et al., 1994; Erne-Brand et al., 1999; Dwyer et al., 2010) and pilot experiments in our laboratory. Behavioral or motor function changes induced by the different treatments were assessed in catheterized rats from all groups by testing the animals’ ability to stand and walk in a normal posture, as proposed elsewhere (Chen and Pan, 2001).

#### 2.2.3 Surgical procedures for electrophysiological experiments

Animals were anesthetized with urethane (2 g/kg), and then an intratracheal cannula was inserted for artificial ventilation (65–75 strokes/min; model Ro Vent^®^, Kent Scientific Corp. United States). Subsequently, animals were mounted onto a stereotaxic frame (Kopf Instruments, United States) and secured in a spinal cord unit frame. The lumbar vertebrae were fixed to improve stability at the recording site to perform a laminectomy to expose the L2-L4 spinal cord segments. The dura was carefully removed, and the exposed spinal cord was covered with isotonic saline to avoid desiccation. The animals were not paralyzed, and we did not observe any withdrawal response during peripheral electrical stimulation. End-tidal CO_2_ was monitored with a Capstar-100 End-tidal CO_2_ analyzer (CWE Inc., United States) and kept between 3.0% and 3.5% by adjusting the stroke volume. Core body temperature was maintained at 38°C using a circulating water pad.

#### 2.2.4 Extracellular unitary recordings and study design

Extracellular unitary recordings were made using seven quartz-Pt/W microelectrodes (impedance 4–7 MΩ) mounted in a multichannel microdrive with an integrated preamplifier. This multi-electrode system was manipulated with the 7-channel version of the fiber-electrode manipulator “System Eckhorn” using Eckhorn Matrix multiuser software (Thomas Recording GmbH, Germany). The microelectrodes were lowered (400–900 μm from the surface) in small steps (2–5 μm/s) into the superficial laminae of the left dorsal horn segments to search for single-unit discharges. For each recorded cell, the specific somatic receptive field (RF) was located by gently tapping on the entire ipsilateral glabrous surface of the hind paw. When we found a RF, electrical stimulation using an S88 stimulator (Grass Instruments Co., United States) was then applied. In this case, two needles (27 G) attached to a stimulus isolator unit (PSIU6 model, Grass Instruments Co., United States) were subcutaneously inserted into the RF of the recorded neuron. The electrical stimulation was then conducted and consisted of 20 stimuli at 0.2 Hz with a 1 ms pulse duration at 1.5 times the threshold intensity (0.1–3 mA) required to evoke a C-fiber discharge. It is interesting to note that using this protocol, the spikes associated with activation of Aβ-, Aδ-, C-fibers, and post-discharge can be observed, but the wind-up was not consistently elicited.

The extracellular neuronal activity induced by electrical stimulation of the RF was amplified ×100 (1700 Differential AC amplifier, A-M Systems, United States), digitalized, and discriminated using CED hardware and Spike 2 software (v5.15; Cambridge Electronic Design, United Kingdom). Raw and discriminated signals were fed through an audio monitor (model 3300, A-M Systems, United States) and displayed on an oscilloscope (TBS1064, Tektronix Inc., United States). Waveforms and recorded spike trains were stored on a hard drive for offline analysis. Evoked activities of the spinal dorsal horn WDR neurons were recorded and analyzed as cumulative frequency and peri-stimulus time histograms (PSTH) to detect the occurrence of neuronal responses. On this basis, the stimulating threshold to evoke action potentials and their frequency of occurrence, resulting from the stimulation of the peripheral RF on the hind paw, were attributed to the recruitment of Aβ-, Aδ-, and C-fibers. Considering the distance between the RF and the recording electrode, the peak latencies observed correspond to peripheral conduction velocities within the Aβ-, (0–20 ms), Aδ- (21–90 ms), C-fibers (90–350 ms), and post-discharge (350–800 ms) (Urch & Dickenson, 2003; González-Hernández et al., 2019; Gamal-Eltrabily et al., 2020). By using this protocol, we exclusively recorded WDR cells, a type of second-order neurons receiving input concomitantly from non-nociceptive (Aβ-type) and nociceptive (Aδ- and C-type) fibers; in addition, post-discharge was also analyzed.

Indeed, during the search of WDR cells responding to peripheral tactile and electrical RF stimulation, some neurons can be classified as only tactile sensitive and, in minor proportion, nociceptive specific; but these types of cells were not further analyzed. Thus, the number of action potentials in response to 20 RF stimuli was compared before (basal, t = 0) and after treatments. In these experiments, a control group without treatment was used (*n* = 5).

Accordingly, the neuronal evoked responses were evaluated immediately after (basal; t = 0) clonidine (1 or 10 nmol; *n* = 5 cells, each) or vehicle (isotonic saline, 0.9% NaCl; *n* = 5 cells) administration and at 10, 20, 30, 40, 50, and 60-min post-treatment. Since in the formalin test, clonidine-induced flinching inhibition was reversed by BRL 44408 but not by imiloxan or JP 1302, the role of the α_2A_-adrenoceptor was evaluated by spinal administration of BRL 44408 (1 nmol; *n* = 5 cells; given 10 min prior clonidine). Furthermore, considering that JP 1302 exhibited antinociceptive effects in the formalin test, we tested the effects of this α_2C_-adrenoceptor antagonist (1 or 10 nmol; *n* = 5 cells, each) on the electrophysiological responses of WDR cells. The vehicle and compounds were given at the spinal cord level (topical) in a total volume of 10 μl using a Hamilton^®^ syringe.

### 2.3 Drugs

This study used the following compounds besides the anesthetics (ketamine, xylazine, and urethane). From Sigma Chemical Co., (United States): 1) clonidine hydrochloride (CAS number: 4205-91-8), 2) 2-[2H-(1-methyl-1,3-dihydroisoindole) methyl]-4,5-dihydroimidazole maleate (BRL 44408; CAS number: 118343-19-4), 3) 2-(1-ethyl-2-indazoyl) methyl-1,4-benzodioxan hydrochloride (imiloxan; CAS number: 81167-22-8) and 4) (-)-bicuculline methiodide (CAS number: 40709-69-1). The N-[4-(4-methyl-1-piperazinyl)phenyl]-9-acridinamine dihydrochloride (JP-1302; CAS number: 1259314-65-2) was acquired from Tocris Ltd., (United States). The doses of clonidine, BRL 44408, imiloxan, and JP 1302 refer to their free base, whereas those of the bicuculline refer to their salt (methiodide). Furthermore, in Table 1, the doses in nmol and μg is given. All drugs were dissolved in a physiological saline solution (0.9% NaCl).

### 2.4 Data presentation and statistical analysis

The data in the figures are presented as mean ± S.E.M., (standard error of the mean). In all cases and before performing a parametric statistical analysis, we checked for normality using the Shapiro-Wilk test (*p* > 0.05). In the formalin test, curves were constructed by plotting the number of flinches as a function of time. The area under the number of flinches against time curves (AUC), an expression of the duration and intensity of the effect, was calculated by the trapezoidal rule, and a one-way analysis of variance (ANOVA) was performed.

For electrophysiological experiments, a baseline neuronal response was established after an identified neuron had a ≤10% variation in the neuronal responses induced by RF stimulation during five consecutive tests (5 min between each trial). The number of basal (t = 0) evoked potential (total spikes and number of Aβ-, Aδ-, C-fibers and post-discharge) in the different experimental groups were analyzed; since the normality test failed, a Kruskal-Wallis one-way analysis of variance (ANOVA) on ranks was performed (see Table 2). On this basis, and to normalize the data, the evoked potentials induced by electrical stimulation of the paw were expressed as a percentage change from the respective baseline. Thus, the baseline value refers to the evoked neuronal response before spinal treatment with clonidine or antagonists. To evaluate the stability of the recorded neurons (only for the control group and vehicle group) across the 60-min time frame, we used a one-way repeated measures ANOVA. The difference in neuronal activity evoked within one group of animals before and after treatments was compared using a two-way repeated-measures ANOVA. In addition, the temporal course was adjusted to obtain global neuronal activity due to the treatment (box and whisker plots); in this case, a one-way ANOVA was performed. The ordinary one-way ANOVA was followed (if applicable) by the Newman-Keuls *post-hoc* test, whereas in the case of the two-way repeated-measures ANOVA, Holm-Sidak’s multiple comparison test was followed. Furthermore, sphericity was not assumed in the case of repeated measures ANOVAs, and corrections to degrees of freedom were made according to the Greenhouse-Geisser method. Differences were considered statistically significant when *p* < 0.05. Graphs and statistical analysis were done using GraphPad Prism V6.0 software (United States). Complete statistical analysis is detailed in Tables 2, 3.

**TABLE 2:** Mean action potentials elicited (±s.e.m.) by twenty electrical stimuli at the basal time (t = 0) in the different experimental groups. Since the normality test failed (Shapiro-Wilk), a Kruskal-Wallis one-way ANOVA on ranks was performed to compare the action potential elicited by the different treatments.

	Control (*n* = 5)	Vehicle (*n* = 5)	Clonidine 1 nmol (*n* = 5)	Clonidine 10 nmol (*n* = 5)	Cloni (10) + BRL 44408 (*n* = 5)	JP 1301 1 nmol (*n* = 5)	JP 1301 10 nmol (*n* = 5)	ANOVA on ranks
Total APs	502.4 ± 80.5	724.6 ± 194.2	762.8 ± 143.4	634.2 ± 130.3	709.2 ± 209.1	617.8 ± 93.5	387.5 ± 92.0	χ^2^ = 4.535 *p* = 0.605
Aβ-fibers	131.8 ± 8.5	123 ± 17.5	134.8 ± 14.7	120.6 ± 20.3	141 ± 26.8	122 ± 14.31	116.0 ± 26.9	χ^2^ = 0.872 *p* = 0.990
Aδ-fibers	38.4 ± 10.6	60 ± 19.0	49.2 ± 14.5	65 ± 7.78	75.6 ± 22.3	70.2 ± 6.8	43.5 ± 10.2	χ^2^ = 7.024 *p* = 0.319
C-fibers	226.4 ± 35.6	363.20 ± 115.0	361.2 ± 101.0	316.4 ± 83.4	342 ± 119.5	293.4 ± 65.2	175.6 ± 57.0	χ^2^ = 1.1 *p* = 0.954
Post-discharge	105.8 ± 39.9	178.4 ± 60.4	127.60 ± 38.7	132.2 ± 40.6	150.6 ± 61.8	132.2 ± 33.9	42.3 ± 12.7	χ^2^ = 5.809 *p* = 0.445

**TABLE 3:** Ordinary one-way or two-way repeated-measures analysis of variance (ANOVA) with their respective *post hoc* comparison for each figure.

Fig	Test	*Post hoc* comparison
2	Ordinary one-way ANOVA	Newman–Keuls multiple comparison test
2A	Tx: F_(3, 16)_ = 19.82; *p* < 0.001	C vs. Cloni[0.1], *p <* 0.001; C vs. Cloni[1], *p <* 0.001; C vs. Cloni[10], *p <* 0.001
2C	Tx: F_(3, 16)_ = 21.35; *p* < 0.001	C vs. Cloni[0.1], *p <* 0.001; C vs. Cloni[1], *p <* 0.001; C vs. Cloni[10], *p <* 0.001
**3**	Ordinary one-way ANOVA	Newman–Keuls multiple comparison test
3B	Tx: F_(5, 24)_ = 20.09; *p* < 0.001	C vs. V + Cloni, *p* < 0.001; C vs. BRL[0.1]+Cloni, *p* < 0.001; C vs. BRL[1]+Cloni, *p* = 0.006; C vs. Imi[10]+Cloni, *p* < 0.001; C vs. JP[1]+Cloni, *p* < 0.001
3C	Tx: F_(5, 24)_ = 12.4; *p* < 0.001	C vs. V + Cloni, *p* < 0.001; C vs. BRL[0.1]+Cloni, *p* < 0.003; C vs. BRL[1]+Cloni, *p =* 0.402; C vs. Imi[10]+Cloni, *p* = 0.001; C vs. JP[1]+Cloni, *p* < 0.001
3E	Tx: F_(4, 20)_ = 4.83; *p* = 0.007	C vs. BRL[0.1], *p* = 0.211; C vs. BRL[1], *p* = 0.205; C vs. Imi[10], *p* = 0.869; C vs. JP[1], *p* = 0.009
3F	Tx: F_(4, 20)_ = 3.38; *p* = 0.029	C vs. BRL[0.1], *p* = 0.831; C vs. BRL[1], *p* = 0.328; C vs. Imi[10], *p* = 0.932; C vs. JP[1], *p* = 0.046
**6**		
6B	Two-way RM ANOVA	Holm–Sidak’s multiple comparison test
	Interaction: F_(24, 120)_ = 0.85;*p* = 0.664	Basal: C vs. V, *p* = 0.061; C vs. Cloni[1], *p* = 0.061; C vs. Cloni[10], *p* = 0.055; C vs. Cloni[10]+BRL, *p* = 0.093 min 10: C vs. V, *p* = 0.132; C vs. Cloni[1], *p =* 0.035; C vs. Cloni[10], *p* = 0.096; C vs. Cloni[10]+BRL, *p* = 0.096
	Time: F_(2.53, 50.57)_ = 2.79; *p* = 0.059	min 20: C vs. V, *p* = 0.512; C vs. Cloni[1], *p* = 0.271; C vs. Cloni[10], *p* = 0.271; C vs. Cloni[10]+BRL, *p* = 0.422
	Tx: F_(4, 20)_ = 2.23; *p* = 0.1023	min 30: C vs. V, *p* = 0.797; C vs. Cloni[1], *p =* 0.302; C vs. Cloni[10], *p* = 0.721; C vs. Cloni[10]+BRL, *p* = 0.721
		min 40: C vs. V, *p* = 0.893; C vs. Cloni[1], *p* = 0.359; C vs. Cloni[10], *p* = 0.595; C vs. Cloni[10]+BRL, *p* = 0.673
		min 50: C vs. V, *p* = 0.626; C vs. Cloni[1], *p* = 0.201; C vs. Cloni[10], *p* = 0.604; C vs. Cloni[10]+BRL, *p* = 0.626
		min 60: C vs. V, *p* = 0.947; C vs. Cloni[1], *p* = 0.829; C vs. Cloni[10], *p* = 0.829; C vs. Cloni[10]+BRL, *p* = 0.837
6C	Two-way RM ANOVA	Holm–Sidak’s multiple comparison test
	Interaction: F_(24, 120)_ = 2.35;*p* = 0.001	Basal: C vs. V, *p* = 0.282; C vs. Cloni[1], *p* = 0.122; C vs. Cloni[10], *p* = 0.547; C vs. Cloni[10]+BRL, *p* = 0.074 min 10: C vs. V, *p* = 0.593; C vs. Cloni[1], *p =* 0.431; C vs. Cloni[10], *p* = 0.289; C vs. Cloni[10]+BRL, *p* = 0.445 min 20: C vs. V, *p* = 0.766; C vs. Cloni[1], *p* = 0.042; C vs. Cloni[10], *p* = 0.022; C vs. Cloni[10]+BRL, *p* = 0.058
	Time: F_(3.698, 73.96)_ = 5.25; *p* = 0.001	min 30: C vs. V, *p* = 0.223; C vs. Cloni[1], *p* = 0.033; C vs. Cloni[10], *p* = 0.005; C vs. Cloni[10]+BRL, *p* = 0.096
	Tx: F_(4, 20)_ = 12.39; *p* < 0.001	min 40: C vs. V, *p* = 0.525; C vs. Cloni[1], *p* = 0.021; C vs. Cloni[10], *p* = 0.002; C vs. Cloni[10]+BRL, *p* = 0.153
		min 50: C vs. V, *p* = 0.412; C vs. Cloni[1], *p* = 0.006; C vs. Cloni[10], *p <* 0.001; C vs. Cloni[10]+BRL, *p* = 0.032
		min 60: C vs. V, *p* = 0.872; C vs. Cloni[1], *p* = 0.012; C vs. Cloni[10], *p* = 0.012; C vs. Cloni[10]+BRL, *p* = 0.872
6D	Two-way RM ANOVA	Holm–Sidak’s multiple comparison test
	Interaction: F_(24, 120)_ = 2.79; *p* < 0.001	Basal: C vs. V, *p* = 0.866; C vs. Cloni[1], *p* = 0.866; C vs. Cloni[10], *p* = 0.866; C vs. Cloni[10]+BRL, *p* = 0.866 min 10: C vs. V, *p* = 0.287; C vs. Cloni[1], *p =* 0.056; C vs. Cloni[10], *p* = 0.033; C vs. Cloni[10]+BRL, *p* = 0.287
	Time: F_(3.3, 66.0)_ = 3.52; *p* = 0.017	min 20: C vs. V, *p* = 0.079; C vs. Cloni[1], *p* = 0.165; C vs. Cloni[10], *p* = 0.011; C vs. Cloni[10]+BRL, *p* = 0.165
	Tx: F_(4, 20)_ = 6.29; *p* < 0.002	min 30: C vs. V, *p* = 0.153; C vs. Cloni[1], *p* = 0.153; C vs. Cloni[10], *p* = 0.010; C vs. Cloni[10]+BRL, *p* = 0.107
		min 40: C vs. V, *p* = 0.037; C vs. Cloni[1], *p* = 0.126; C vs. Cloni[10], *p* = 0.018; C vs. Cloni[10]+BRL, *p* = 0.008
		min 50: C vs. V, *p* = 0.462; C vs. Cloni[1], *p* = 0.462; C vs. Cloni[10], *p* = 0.003; C vs. Cloni[10]+BRL, *p* = 0.046
		min 60: C vs. V, *p* = 0.991; C vs. Cloni[1], *p* = 0.861; C vs. Cloni[10], *p* = 0.047; C vs. Cloni[10]+BRL, *p* = 0.879
6E	Two-way RM ANOVA	Holm–Sidak’s multiple comparison test
	Interaction: F_(24, 104)_ = 1.72; p = 0.03	Basal: C vs. V, p = 0.922; C vs. Cloni[1], p = 0.922; C vs. Cloni[10], p = 0.922; C vs. Cloni[10]+BRL, p = 0.851 min 10: C vs. V, p = 0.906; C vs. Cloni[1], p = 0.388; C vs. Cloni[10], p = 0.102; C vs. Cloni[10]+BRL, p = 0.962
	Time: F_(4.038, 70.0)_ = 0.59; p = 0.67	min 20: C vs. V, p = 0.933; C vs. Cloni[1], p = 0.933; C vs. Cloni[10], p = 0.059; C vs. Cloni[10]+BRL, p = 0.933
	Tx: F_(4, 20)_ = 4.62; p < 0.008	min 30: C vs. V, p = 0.819; C vs. Cloni[1], p = 0.819; C vs. Cloni[10], p = 0.019; C vs. Cloni[10]+BRL, p = 0.619
		min 40: C vs. V, p = 0.995; C vs. Cloni[1], p = 0.995; C vs. Cloni[10], p = 0.004; C vs. Cloni[10]+BRL, p = 0.995
		min 50: C vs. V, p = 0.875; C vs. Cloni[1], p = 0.763; C vs. Cloni[10], p = 0.096; C vs. Cloni[10]+BRL, p = 0.763
		min 60: C vs. V, p = 0.966; C vs. Cloni[1], p = 0.966; C vs. Cloni[10], p = 0.148; C vs. Cloni[10]+BRL, p = 0.963
6F	Ordinary one-way ANOVA	
	Tx: F_(4, 20)_ = 1.385; *p* = 0.275	
6G	Ordinary one-way ANOVA	Newman–Keuls multiple comparison test
	Tx: F_(4, 20)_ = 12.974; *p <* 0.001	C vs. V, *p* = 0.4; C vs. Cloni[1], *p <* 0.001; C vs. Cloni[10], *p <* 0.001; C vs. Cloni[10]+BRL, *p* = 0.1
6H	Ordinary one-way ANOVA	Newman–Keuls multiple comparison test
	Tx: F_(4, 20)_ = 6.413; *p* = 0.002	C vs. V, *p* = 0.2; C vs. Cloni[1], *p =* 0.2; C vs. Cloni[10], *p <* 0.001; C vs. Cloni[10]+BRL, *p* = 0.9
6I	Ordinary one-way ANOVA	Newman–Keuls multiple comparison test
	Tx: F_(4, 20)_ = 4.169; *p* = 0.013	C vs. V, *p* = 0.9; C vs. Cloni[1], *p =* 0.9; C vs. Cloni[10], *p =* 0.018; C vs. Cloni[10]+BRL, *p* = 0.8
**7**		
7C	Two-way RM ANOVA	Holm–Sidak’s multiple comparison test
	Interaction: F_(18, 96)_ = 1.49; *p* = 0.109	Basal: C vs. V, *p* = 0.061; C vs. JP[1], *p* = 0.099; C vs. JP[10], *p* = 0.099 min 10: C vs. V, *p* = 0.246; C vs. JP[1], *p* = 0.246; C vs. JP[10], *p* = 0.620
	Time: F_(2.8, 44.76)_ = 2.35; *p* = 0.089	min 20: C vs. V, *p* = 0.526; C vs. JP[1], *p* = 0.526; C vs. JP[10], *p* = 0.526
	Tx: F_(3, 16)_ = 0.782; *p* = 0.522	min 30: C vs. V, *p* = 0.941; C vs. JP[1], *p* = 0.744; C vs. JP[10], *p* = 0.941
		min 40: C vs. V, *p* = 0.981; C vs. JP[1], *p* = 0.907; C vs. JP[10], *p =* 0.981
		min 50: C vs. V, *p* = 0.735; C vs. JP[1], *p =* 0.735; C vs. JP[10], *p =* 0.735
		min 60: C vs. V, *p* = 0.947; C vs. JP[1], *p* = 0.615; C vs. JP[10], *p* = 0.799
7D	Two-way RM ANOVA	Holm–Sidak’s multiple comparison test
	Interaction: F_(18, 96)_ = 1.63; *p* = 0.67	Basal: C vs. V, *p* = 0.213; C vs. JP[1], *p* = 0.213; C vs. JP[10], *p* = 0.136 min 10: C vs. V, *p* = 0.645; C vs. JP[1], *p* = 0.645; C vs. JP[10], *p* = 0.243
	Time: F_(3.39, 54.27)_ = 0.99; *p* = 0.409	min 20: C vs. V, *p* = 0.766; C vs. JP[1], *p* = 0.255; C vs. JP[10], *p* = 0.199
	Tx: F_(3, 16)_ = 2.351; *p* = 0.111	min 30: C vs. V, *p* = 0.443; C vs. JP[1], *p* = 0.443; C vs. JP[10], *p* = 0.443
		min 40: C vs. V, *p* = 0.665; C vs. JP[1], *p* = 0.223; C vs. JP[10], *p* = 0.665
		min 50: C vs. V, *p* = 0.411; C vs. JP[1], *p =* 0.117; C vs. JP[10], *p =* 0.055
		min 60: C vs. V, *p* = 0.739; C vs. JP[1], *p* = 0.143; C vs. JP[10], *p* = 0.739
7E	Two-way RM ANOVA	Holm–Sidak’s multiple comparison test
	Interaction: F_(18, 96)_ = 1.60; *p* = 0.074	Basal: C vs. V, *p* = 0.774; C vs. JP[1], *p* = 0.774; C vs. JP[10], *p* = 0.349 min 10: C vs. V, *p* = 0.287; C vs. JP[1], *p* = 0.448; C vs. JP[10], *p* = 0.201
	Time: F_(2.37, 37.95)_ = 0.415; *p* = 0.697	min 20: C vs. V, *p* = 0.079; C vs. JP[1], *p* = 0.233; C vs. JP[10], *p* = 0.084
	Tx: F_(3, 16)_ = 0.829; *p* = 0.497	min 30: C vs. V, *p* = 0.221; C vs. JP[1], *p* = 0.287; C vs. JP[10], *p* = 0.287
		min 40: C vs. V, *p* = 0.055; C vs. JP[1], *p* = 0.338; C vs. JP[10], *p* = 0.338
		min 50: C vs. V, *p* = 0.462; C vs. JP[1], *p =* 0.464; C vs. JP[10], *p =* 0.464
		min 60: C vs. V, *p* = 0.991; C vs. JP[1], *p* = 0.922; C vs. JP[10], *p* = 0.990
7F	Two-way RM ANOVA	Holm–Sidak’s multiple comparison test
	Interaction: F_(18, 91)_ = 2.31; p = 0.005	Basal: C vs. V, p = 0.922; C vs. JP[1], p = 0.946; C vs. JP[10], p = 0.922 min 10: C vs. V, p = 0.972; C vs. JP[1], p = 0.972; C vs. JP[10], p = 0.972
	Time: F_(2.011, 30.5)_ = 1.31; p = 0.286	min 20: C vs. V, p = 0.840; C vs. JP[1], p = 0.840; C vs. JP[10], p = 0. 840
	Tx: F_(3, 16)_ = 0.916; p = 0.456	min 30: C vs. V, p = 0.666; C vs. JP[1], p = 0.652; C vs. JP[10], p = 0. 652
		min 40: C vs. V, p = 0.824; C vs. JP[1], p = 0.404; C vs. JP[10], p = 0.506
		min 50: C vs. V, p = 0.733; C vs. JP[1], p = 0.743; C vs. JP[10], p = 0.733
		min 60: C vs. V, p = 0.595; C vs. JP[1], p = 0.595; C vs. JP[10], p = 0.421
7G	Ordinary one-way ANOVA	Newman–Keuls multiple comparison test
	Tx: F_(3, 16)_ = 4.308; *p* = 0.021	C vs. JP, *p* = 0.013; C vs. JP + Bicu[0.3], *p =* 0.218; C vs. Bicu[0.3], *p =* 0.181
7H	Ordinary one-way ANOVA	Newman–Keuls multiple comparison test
	Tx: F_(4, 20)_ = 6.413; *p* = 0.002	C vs. JP, *p* = 0.047; C vs. JP + Bicu[0.3], *p =* 0.638; C vs. Bicu[0.3], *p =* 0.9

Abbreviations: Control (C); Clonidine (Cloni); BRL 44408 (BRL); Imiloxan (Imi); JP 1302 (JP); Bicuculline (Bicu).

## 3 Results

### 3.1 Intrathecal clonidine inhibits flinching behavior induced by formalin

**FIGURE 2:**
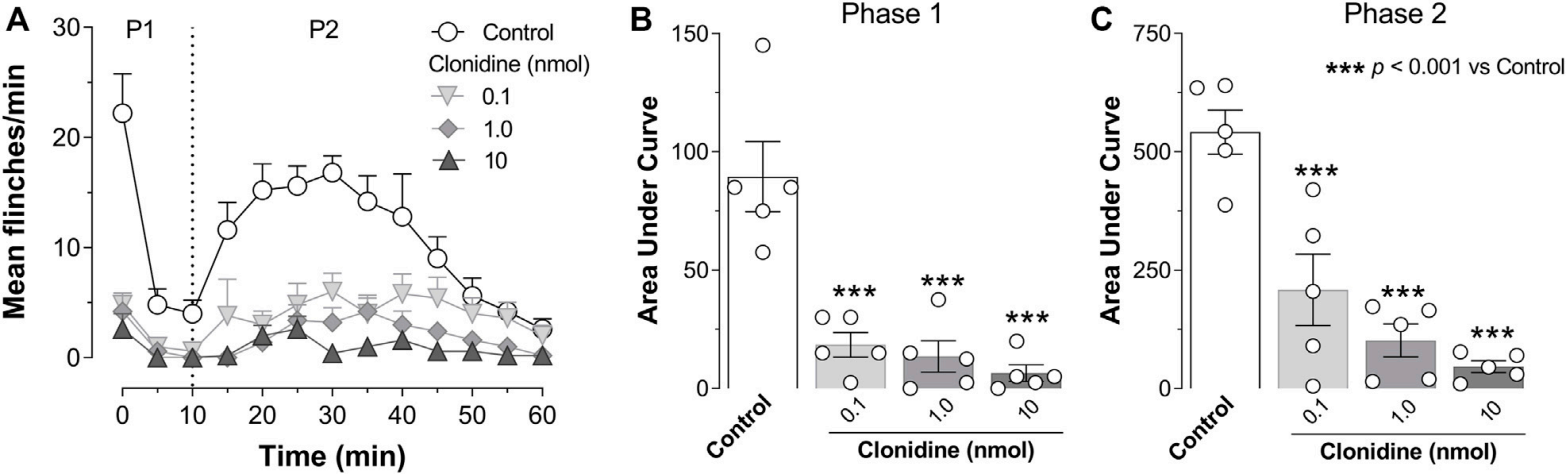
Intrathecal (i.t.) clonidine (α_2A/2B/2C_-adrenoceptor agonist) inhibits behavioral nociception. **(A)** Flinching number over time during phase I (P1) and phase II (P2) of the mean number per minute of flinches observed after i.t., clonidine (0.1, 1, and 10 nmol; *n* = 5 each dose) in rats submitted to the 1% formalin test (50 μl, s.c.). **(B,C)** show the time course data expressed as the area under the mean number of flinches against the time curve (AUC). Clonidine reduced AUC values during both phases (P1, P2), indicating an antinociceptive effect. Clonidine was given 10 min prior to formalin injection.

Subcutaneous (s.c.) formalin injection into the right hind paw produced a typical flinching behavior characterized by a biphasic time course (Figure 2A; control curve). Phase I of the nociceptive response began immediately after formalin injection and gradually declined (≈10 min). Then, phase II started about 15 min after formalin injection and lasted 1 h. I.t administration of clonidine (0.1, 1, and 10 nmol) inhibited the formalin-induced flinching behavior during phases I and II (Figures 2A–C).

### 3.2 Effect of α_2A_- (BRL 44408), α_2B_- (imiloxan), and α_2C_- (JP 1302) adrenoceptor antagonists in the clonidine-induced behavioral antinociception

**FIGURE 3:**
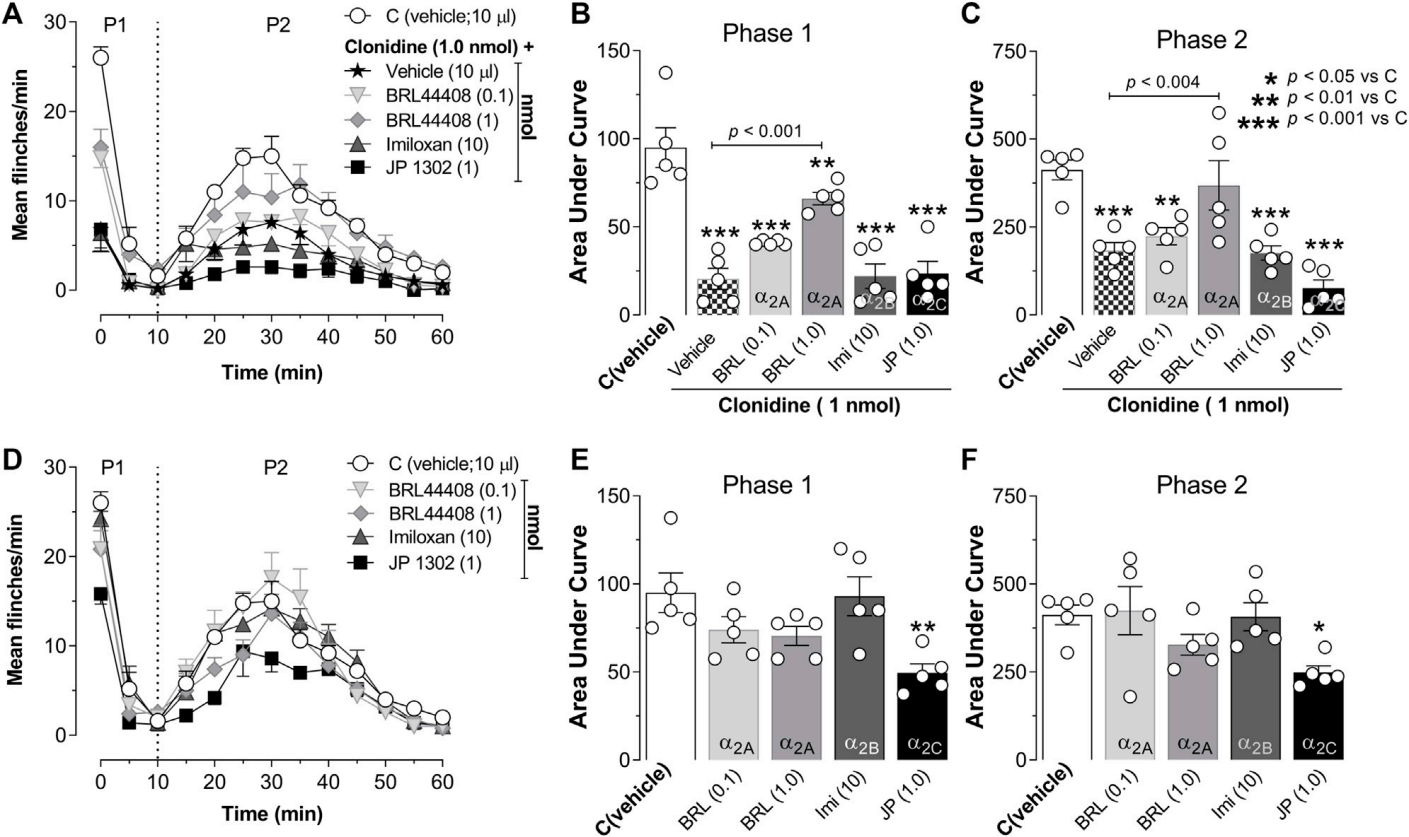
Role of spinal α_2A_-adrenoceptors in the clonidine-induced behavioral antinociception and the antinociceptive effect of JP 1302 (α_2C_-adrenoceptor antagonist). **(A)** shows the effect of intrathecal (i.t.) injection of vehicle (10 μl; isotonic saline solution) or α_2A/2B/2C_-adrenoceptor antagonists on the clonidine-induced inhibition of the flinches induced by formalin (1%; 50 μl, s.c.) during phase I (P1) and phase II (P2). Vehicle, BRL 44408 (0.1 or 1 nmol; an α_2A_-adrenoceptor antagonist; *n* = 5 each dose), imiloxan (10 nmol; an α_2B_-adrenoceptor antagonist; *n* = 5), or JP 1302 (1 nmol; an α_2C_-adrenoceptor antagonist; *n* = 5) were given i.t. 10 min prior clonidine. **(B,C)** represent the data as the area under the mean number of flinches against the curve (AUC) and show that BRL 44408 (1 nmol) inhibited the clonidine-induced antinociception in both phases (P1, P2). During P2, JP 1302 seems to enhance (statistically non-significant, *p* > 0.05) the antinociception induced by clonidine. **(D)** shows the *per se* effect of BRL 44408 (n = 5 each dose), imiloxan (*n* = 5), or JP 1302 (*n* = 5 each dose) in rats submitted to the 1% formalin test. The AUC in **(E,F)** suggest that BRL 44408 and imiloxan do not affect formalin-induced nociception. In contrast, JP 1302 diminishes the AUC values during both phases (P1, P2), suggesting an antinociceptive effect.

As shown in Figure 3A, the antinociception induced by 1 nmol clonidine was attenuated by 1 nmol BRL 44408 and remained unaffected by 0.1 nmol BRL 44408, 10 nmol imiloxan, or 1 nmol JP 1302. In particular, 1 nmol BRL 44408 partially reverted the clonidine effect during phase I (Figure 3B) and abolished the clonidine-induced antinociception during phase II (Figure 3C).

When we tested the *per se* effects induced by the antagonists (Figure 3D), we found that i.t. administration of vehicle (0.9% NaCl solution, 10 µl), BRL 44408 (0.1, 1 nmol) or imiloxan (10 nmol) did not have a statistical difference (see Table 2 for details) on the flinching behavior induced by formalin; in contrast, i.t. JP 1302 significantly reduced the number of flinches during phase I and II (Figures 3E,F).

Since the behavioral experiments showed that clonidine inhibits the formalin-induced nociception *via* α_2A_-adrenoceptor activation, a set of electrophysiological recordings of the second-order WDR dorsal horn spinal cord cells were performed to correlate the behavioral outcome with electrophysiological responses.

### 3.3 General effects of peripheral electrical stimuli on wide dynamic range cell responses

**FIGURE 4:**
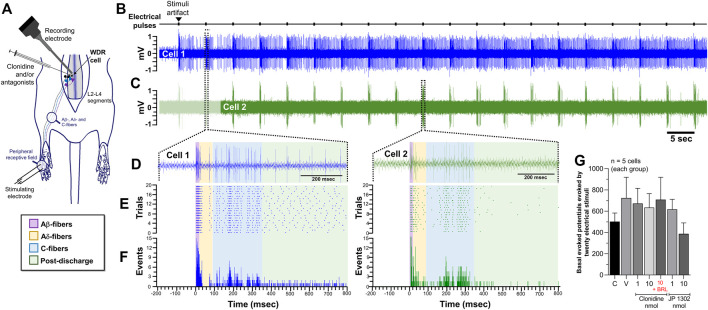
Effect of peripheral electrical stimulation on the WDR cell response. **(A)** Experimental set-up design illustrating the site of electrophysiological recording of WDR cells at spinal L2-L4 segments, and the location, in the ipsilateral paw of the electrical stimulation applied to the receptive field (RF). **(B)** Raw data of twenty stimulus artifacts and **(C)** raw data of consecutive WDR responses to RF stimulation in two different cells (Cell 1 and Cell 2). **(D)** shows the raw tracing of a single second-order WDR neuron cluster response induced by one electrical stimulus-evoked in Cell 1 (left) or Cell 2 (right). **(E)** shows the raster plot and **(F)** peri-stimulus time histograms (PSTH) constructed from twenty WDR neuronal responses to RF stimulation depicting the different fiber components (Aβ-, Aδ-, C-fibers, and post-discharge). Note that, under our experimental condition, no clear wind-up is elicited. **(G)** shows that the basal response, i.e., the total of action potentials evoked at t = 0 in the different experimental groups, is not statistically different (χ^2^ = 4.535; *p* = 0.605).

Figure 4A illustrates the experimental setup used to perform the *in vivo* unitary extracellular recordings of spinal WDR cells. In this figure, the recordings correspond to the baseline neuronal response (t = 0) elicited by twenty electrical pulses given in the paw receptive field. All neurons recorded in the present experiments were found at an average of 700 ± 192 μm from the spinal surface. As illustrated in Figures 4B,C, the peripheral electrical stimulation produces a well-defined and stereotyped WDR cell response. In general, under our parameters of electrical stimulation used (0.2 Hz; 1 ms pulse duration), we observed that although some cells exhibited high post-discharge events (see Cell 1), an inconsistent wind-up was elicited (calculated accordingly to the formula given by Gjerstad et al., 1996). As previously reported, wind-up is considered as a facilitation of neural discharge evoked by repetitive stimulation of primary afferent fibers (Mendell, 1966). This process elicited in WDR cells seems to mainly depend on the frequency stimulation, and in general, frequencies >0.5 Hz are required to consistently produce a wind-up (Mendell, 1966; Dickenson and Sullivan, 1987; Chapman and Dickenson, 1992; Li et al., 1999; You et al., 2003); in this sense, 0.2 Hz seems not to be enough to recruit a facilitatory input leading to wind-up.

Furthermore, at the basal time (t = 0), the mean average evoked potential of all animals tested was 667 ± 29 spikes. Since the evoked potential did not follow a normal distribution along the experimental groups, a non-parametric test was used, and we found no statistically significant difference (see Figure 4G; Table 2 for details). The following results were normalized to perform a parametric statistical analysis.

Since we recorded spinal WDR cells for 60 min, analyzing the effect of time on the neuronal responses was crucial to exclude a time-dependent effect. The one-way ANOVA suggests that no time-dependent changes in neuronal responses occurred during our experimental protocols. Specifically, in the control group, time had no effect on Aδ- (F_(2.73, 10.90)_ = 0.213; *p* = 0.869) or C-fibers (F_(2.09, 8.36)_ = 1.92; *p* = 0.197). Similar results were obtained in the case of vehicle administration [Aδ- (F_(2.68, 10.76)_ = 0.204; *p* = 0.869) and C-fibers (F_(2.27, 9.09)_ = 0.892; *p* = 0.455)].

### 3.4 Spinal clonidine-induced electrophysiological antinociception is reversed by BRL 44408

**FIGURE 5:**
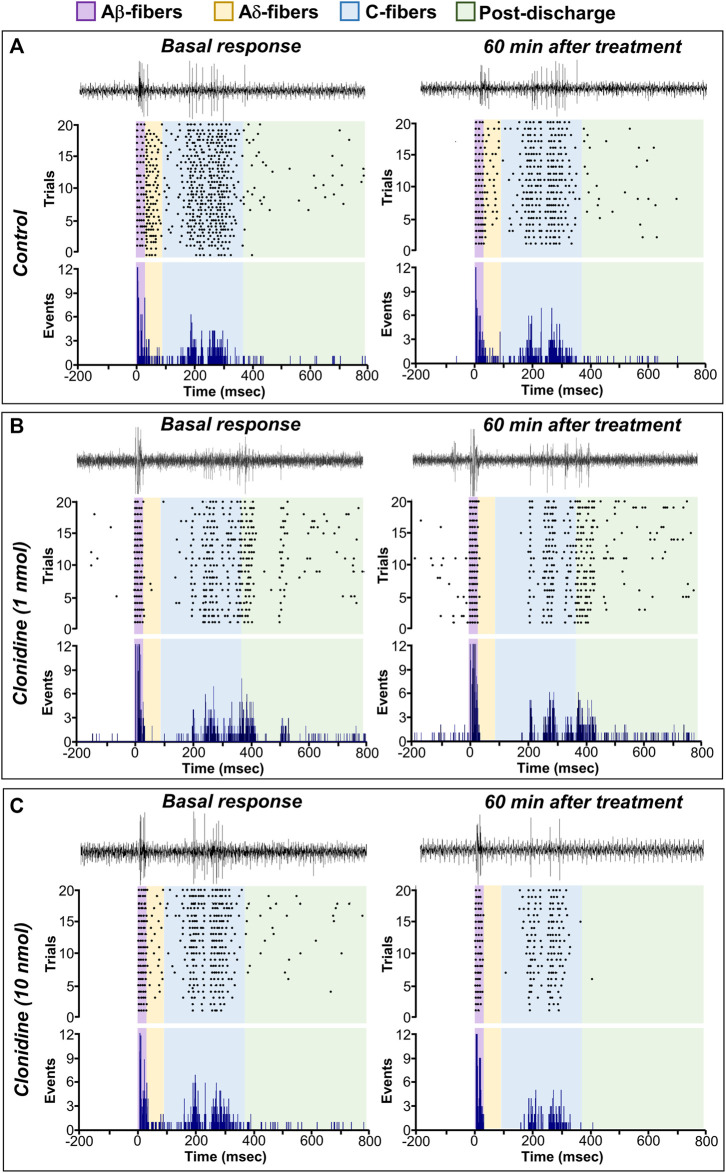
Spinal clonidine selectively blocks the nociceptive neuronal firing of WDR cells. **(A–C)** depict the raw tracing of a single second-order WDR neuron cluster response induced by one electrical stimulus (upper tracing), the raster plot (middle), and peri-stimulus time histograms (PSTH) constructed from 20 WDR responses to receptive field (RF) electrical stimulation before (basal) and after treatment. In accordance with the fiber conduction velocities, the panels in the figure depict the different fiber components (Aβ-, Aδ-, C-fibers, and post-discharge) of the spinal WDR cell response. Note that clonidine (1 and 10 nmol) diminished the neuronal activity; this clonidine-induced inhibition (observed as a diminution of events) is mainly associated with neural action on nociceptive fibers (i.e., Aδ- and C-fibers) and ongoing (post-discharge) activity, whereas the firing of Aβ-fibers remains unaltered.

Figure 5 shows the raw tracing of a single WDR neuron cluster response, the raster plots, and the PSTHs before and after different treatments. Figure 5A shows the PSTHs obtained during the control condition, whereas Figures 5B,C exemplify the neural activity of second-order WDR cells before (basal response) and after clonidine treatment. In contrast to the control group (Figure 5A), spinal clonidine, particularly at 10 nmol (Figure 5C), diminished the neuronal firing responses (events) elicited by the RF electrical stimulation. We must clarify that each PSTH represents the neuronal evoked activity of one WDR cell induced by twenty electrical pulses; these evoked neuronal events could be broken down according to the conduction velocities of the primary afferent fibers (i.e., Aβ-, Aδ-, and C-fibers).

**FIGURE 6:**
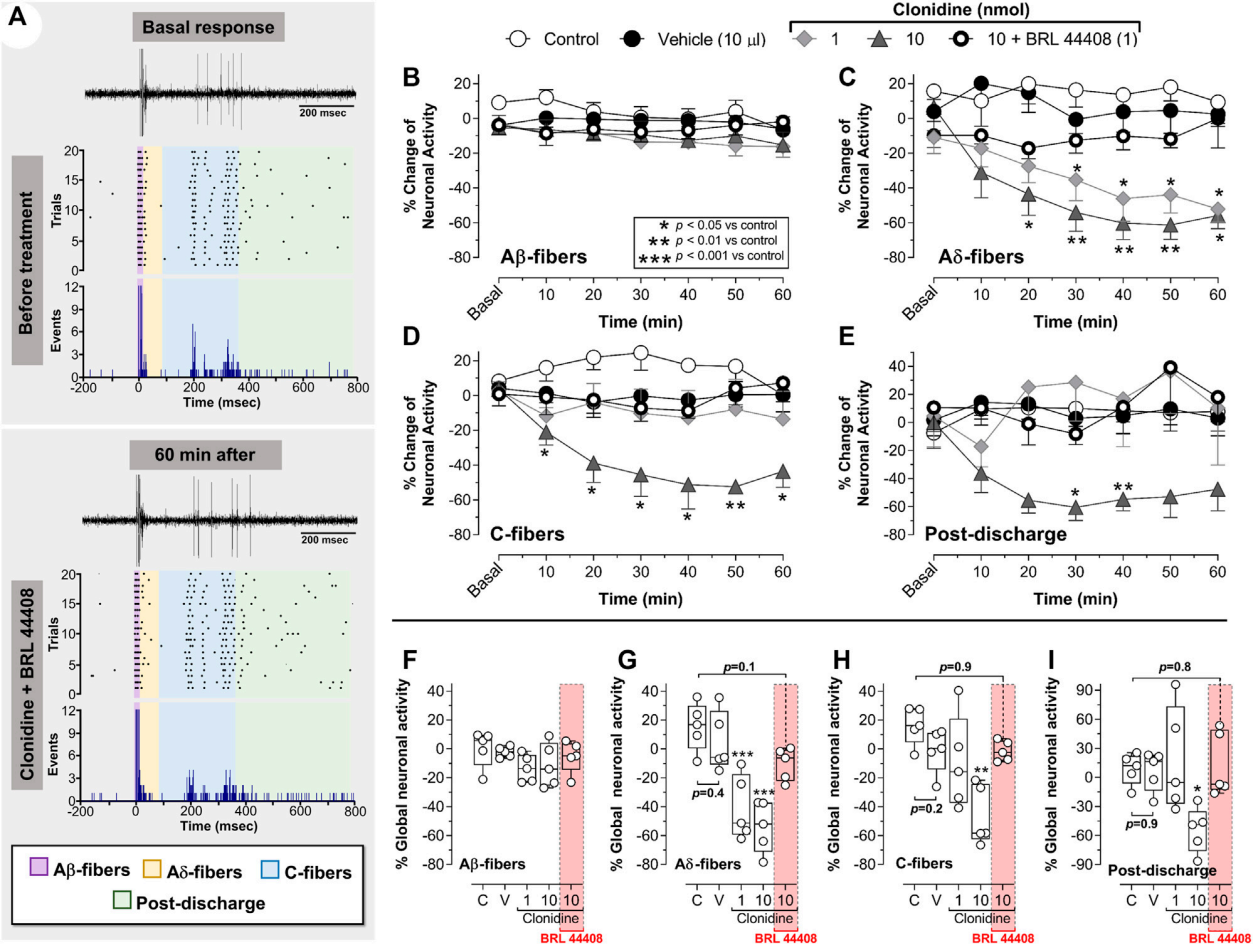
The role of spinal α_2A_-adrenoceptors in clonidine-induced electrophysiological antinociception. In **(A)**, a pair of raw tracings of a single second-order WDR neuron cluster response induced by one electrical stimulus (upper tracing), the raster plot (middle), and peri-stimulus time histogram (PSTH) depicting the effect of BRL 44408 on the clonidine-induced antinociception. Notice that under α_2A_-adrenoceptors blockade with BRL 44408 (1 nmol), the antinociceptive action of clonidine is not elicited. **(B–E)** show the time-course changes in the percentage average of the different fibers activating WDR cell responses and post-discharge elicited by receptive field (RF) electrical stimulation and the effects of spinal clonidine (1 and 10 nmol/10 μl; *n* = 5 each dose). Notice that 1 nmol clonidine only inhibits the neuronal firing of Aδ-fibers **(C)**, while 10 nmol clonidine inhibits the activity of Aδ-fibers **(C),** C-fibers **(D)**, and post-discharge **(E)**. **(F–I)** show the global neuronal activity (obtained from the respective time course figures) of Aβ-, Aδ-, C-fibers, and post-discharge in response to spinal clonidine. Clonidine preferentially blocks the neuronal activity associated with Aδ-, C-fibers, and post-discharge but not Aβ-fibers. On the other hand, blockade of the α_2A_-adrenoceptors with BRL 44408 (1 nmol) inhibits the clonidine-induced electrophysiological antinociception.

In this set of experiments, we found that BRL 44408 reversed the antinociception induced by clonidine. Figure 6A shows an example of a raw tracing of a single WDR neuron cluster response, the raster plots, and PSTH obtained in an animal pre-treated with BRL 44408 before clonidine administration. Note that BRL 44408 blocked the clonidine-induced diminution of WDR neuronal activity.

Upon quantifying the data from the PSTHs, we found that spinal clonidine decreases the firing response elicited by electrical stimuli on the RF (Figures 6B–I). Specifically, clonidine has no substantial effect on the neuronal activity associated with Aβ-fiber activation (Figure 6B), but we observed a potent inhibition of neuronal activity related to the activation of Aδ- (Figure 6C) rather than C-fibers (Figure 6D). This antinociceptive effect started 10 min after clonidine administration, peaked at 40–50 min and lasted up to 60 min as previously reported (Sullivan et al., 1987; Bonnet et al., 1989; Murata et al., 1989). Furthermore, as expected, the clonidine-induced inhibition of nociceptive Aδ- and C-fiber activity was reversed by spinal pre-treatment with BRL 44408 (1 nmol). In addition, as shown in Figure 6E, the post-discharge activity was reduced by 10 nmol clonidine, an effect reversed by BRL44408. Analyzing these results as global neuronal activity (Figures 6F–I), we corroborated that clonidine selectively blocks the neuronal activity associated with the activation of primary nociceptive fibers, and this effect is abolished by a selective α_2A_-adrenoceptor antagonist (BRL 44408). Together, these results highlight the relevance of primary afferent fibers innervating second-order WDR cells in clonidine-induced antinociception.

### 3.5 Spinal JP 1302 does not influence the neuronal activity of second-order wide dynamic range cells

**FIGURE 7:**
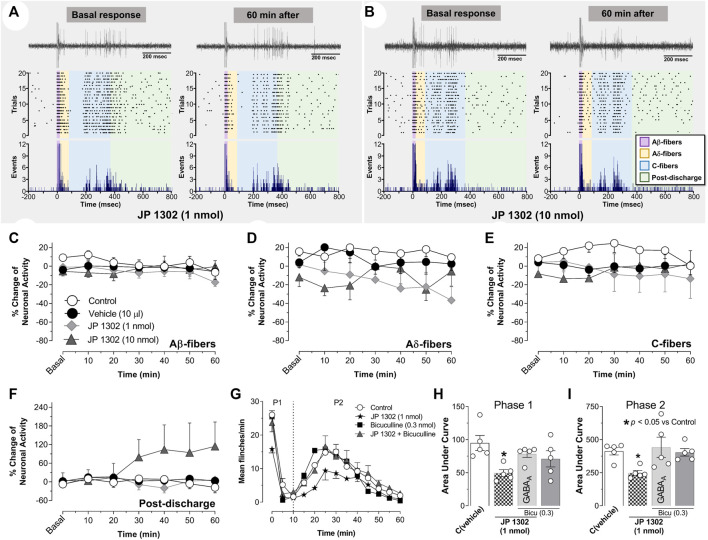
Spinal JP-1302 does not affect wide dynamic range (WDR) cell activity, but behaviorally, it inhibits nociception by increasing GABAergic activity. **(A,B)** depict the peri-stimulus time histogram (PSTH) constructed from 20 WDR responses to receptive field (RF) electrical stimulation before (basal) and after treatment (vehicle or JP 1302); also, in these panels, the raster plot and raw tracing of a single second-order WDR neuron cluster response elicited by one electrical stimulus (upper tracing) are illustrated. In accordance with the fiber conduction velocities, the panels in the figure depict the different fiber components (Aβ-, Aδ-, and C-fibers) of the spinal WDR cell response. Note that JP 1302 seems not to exert an effect on the WDR neuronal activity; indeed, when the data are analyzed as a percent of change of the neuronal activity **(C–F)**, JP 1302 does not impact (statistically) the neuronal firing associated with the activation of Aβ-, Aδ-, C-fibers, and post-discharge. **(G)** shows the effect of intrathecal (i.t.) injection of 0.3 nmol bicuculline (a preferent GABA_A_ receptor blocker) on the JP 1302-induced inhibition of the flinches induced by formalin (1%; 50 μl, s.c.) during phase I (P1) and phase II (P2). Note that bicuculline *per se* did not affect formalin-induced flinching behavior, but this GABA_A_ channel blocker reversed the effect of JP 1302. **(H,I)** represent the data as the area under the mean number of flinches against the curve (AUC) and show that bicuculline inhibited the JP 1302-induced antinociception in both phases (P1, P2).

Based on the behavioral results, where 1 nmol JP 1302 *per se* inhibited the flinches evoked by formalin (Figures 3D–F), we tested the effect of JP 1302 on WDR neuronal activity (Figures 7A–F). The results showed that spinal JP 1302 (1 or 10 nmol) did not affect the peripheral evoked neuronal activity of Aβ-, Aδ-, C-fibers and post-discharge (Figures 7C–F). These results may imply that the mechanisms involved in the antinociception induced by JP 1302 are not mediated by an action on WDR neurons or primary afferent fibers innervating these second-order cells.

### 3.6 JP 1302-induced behavioral antinociception is blocked by spinal bicuculline

On this basis and considering that some evidence suggests that α_2C_-adrenoceptors decrease GABAergic transmission (see discussion section), we hypothesized that the JP 1302-induced behavioral antinociception could be indirectly mediated by a spinal GABAergic mechanism. Accordingly, i.t., administration of bicuculline (0.3 nmol; a GABA_A_ receptor blocker) abolished the behavioral antinociception induced by JP 1302 (1 nmol) in phases I and II (Figures 7G–I). In this case, and as previously reported in the formalin test (Dirig and Yaksh, 1995; Peng et al., 2015; Ryu et al., 2021) bicuculline *per se* does not influence flinching behavior.

## 4 Discussion

### 4.1 General

**FIGURE 8:**
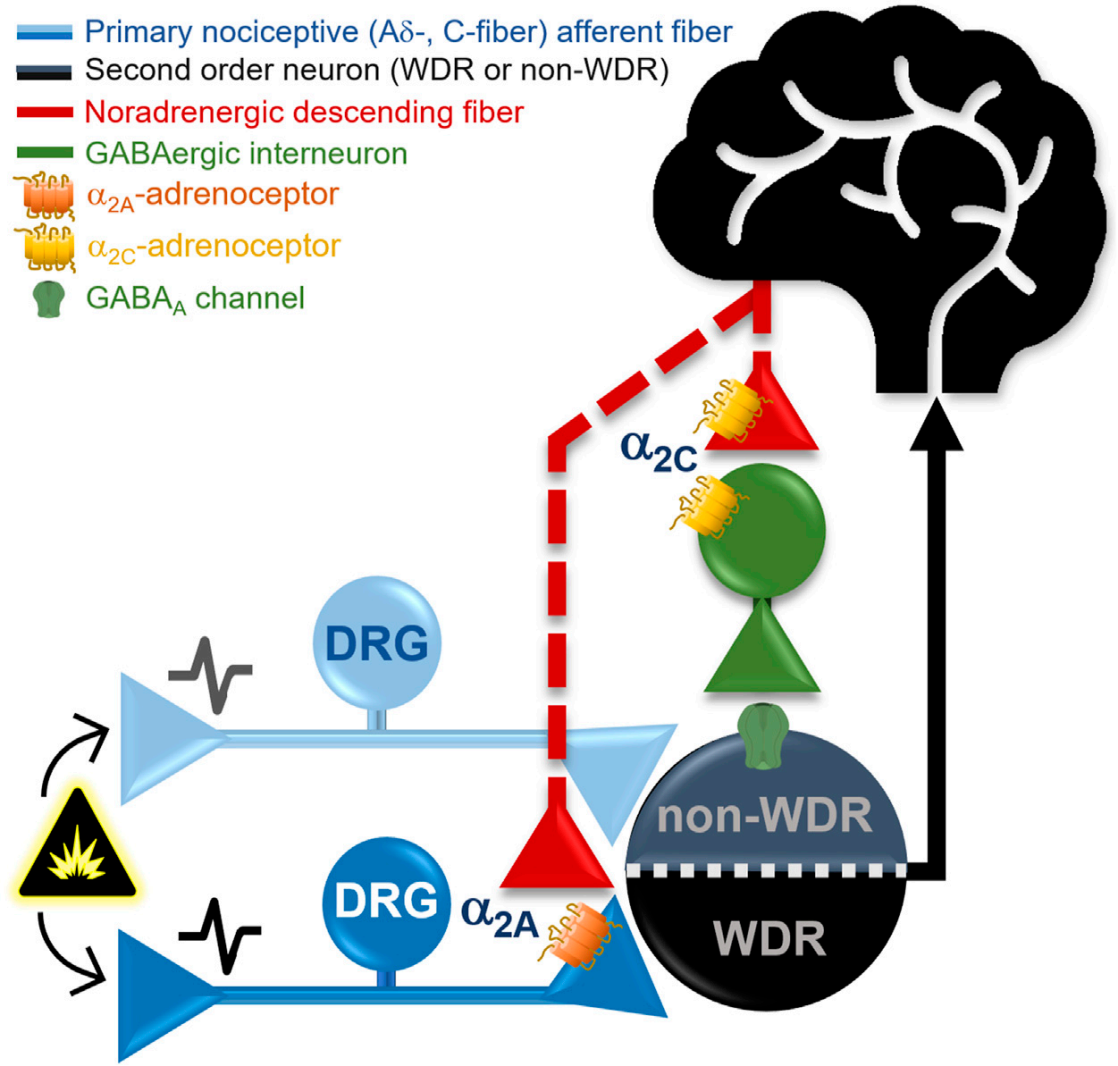
Proposed mechanisms implying a differential role of the α_2A_- and α_2C_-adrenoceptors subtypes in pain modulation at the spinal cord level. In general, α_2_-adrenoceptors are coupled to G_i/o_ proteins; thus, when activated, they could promote the inhibition of neurotransmitter release by inhibiting adenylate cyclase activity and inactivating Ca^2+^ channels and/or facilitation of K^+^ currents. Our behavioral and electrophysiological data support the contention that clonidine (a non-selective α_2A/2B/2C-_adrenoceptor agonist) induces antinociception *via* activation of α_2A-_adrenoceptors, probably located at presynaptic level (in the primary nociceptive Aδ- and C-fibers), causing a diminution of the WDR cell nociceptive firing. Indeed, α_2A-_adrenoceptors activation inhibits tonic (formalin) and acute (electrical) nociceptive input. On the other hand, the selective α_2C-_adrenoceptor antagonist, JP 1302, produces *per se* only behavioral but not electrophysiological antinociception (measured on second WDR cells), suggesting an inhibitory effect on tonic nociception. Since JP 1302-induced antinociception was blocked by bicuculline, a GABAergic mechanism has been resembled. The data could be interpreted as follows: (i) presynaptic activation of α_2A_-adrenoceptors by clonidine inhibits acute and tonic peripheral nociception; (ii) the JP 1302-induced antinociception is not mediated by inhibition of the nociceptive primary afferent fibers activity; and (ii) considering that α_2C_-adrenoceptors can be found in GABAergic neurons, we could suggest that under a tonic nociceptive stimulus (e.g., inflammatory), activation of α_2C_-adrenoceptors play a pronociceptive role. Hence (see Discussion for details), the data support the notion that α_2A_-adrenoceptor are mainly localized in primary nociceptive afferent fibers modulating the activity of WDR cells. In contrast, α_2C_-adrenoceptors seem to be present in spinal interneurons rather than capsaicin-sensitive fibers modulating second-order nociceptive specific cells.

This study was designed to pharmacologically dissect the role of different α_2A/2B/2C_-adrenoceptor subtypes in clonidine-induced antinociception. Using tonic (i.e., formalin test) or acute (i.e., peripheral electrical stimulation) nociceptive stimuli, we show that clonidine-induced antinociception is mediated by α_2A_- but not α_2B/2C_-adrenoceptor activation. Given that in the electrophysiological experiments, clonidine inhibited the neuronal activity associated with electrical activation of Aδ- and C-fibers (but not Aβ-fibers), a presynaptic effect on nociceptive primary afferent fibers (PAFs) is supported. Furthermore, the ongoing firing (post-discharge) was attenuated by clonidine; an effect also reversed by the BRL 44408. Besides, we found that spinal JP 1302 (a selective α_2C_-adrenoceptor antagonist) induced *per se* behavioral but not electrophysiological antinociception, an effect reversed by bicuculline (a preferential GABA_A_ receptor blocker). Apart from the implications discussed below, our data support the notion that: 1) antinociception (acute or tonic) induced by clonidine is mediated by α_2A_-adrenoceptors, whereas 2) pharmacological blockade of α_2C_-adrenoceptors during tonic nociception elicits antinociception, thus, unmasking a potential pronociceptive action of this receptor (Figure 8).

One important thing in the experimental protocols followed was that the effect of clonidine was analyzed during 1 h. This experimental design was based on the long-lasting antinociception induced by clonidine when given by a spinal route, despite the half-life in the spinal cord being ∼30 min (Castro and Eisenach, 1989; Khan et al., 1999). Accordingly, seminal reports have consistently shown that this compound induces a spinal long-lasting (>1 h) analgesia/antinociception peaking after 30 min post-injection (Sullivan et al., 1987; Bonnet et al., 1989; Murata et al., 1989; Khan et al., 1999). At first glance, we could hypothesize that this long-lasting effect could be partly mediated by the engagement of supraspinal mechanisms enhancing a descending inhibitory pathway. Indeed, we must acknowledge that activation of the endogenous noradrenergic descending pathway plays a key role in spinal pain modulation in naïve and neuropathic rodents (Patel et al., 2018). Nevertheless, Murata et al. (1989) showed in WDR cell recordings that antinociception elicited by spinal clonidine was unaffected in animals spinally transected. Consequently, it seems that the effect of this ligand (when given spinally) mainly relies upon spinal cord level.

### 4.2 Spinal clonidine inhibits the behavioral and electrophysiological nociception by α_2A_ rather than α_2B/2C_-adrenoceptor activation

Our data showed that i.t., clonidine reduces the number of flinches evoked by 1% formalin (Figure 2). This effect is related to spinal α_2_-adrenoceptor activation (Pertovaara, 2013; Llorca-Torralba et al., 2016; Bahari and Meftahi, 2019). Since pretreatment with BRL 44408 (but not imiloxan or JP 1302) reversed the clonidine-induced behavioral antinociception, the role of α_2A_-adrenoceptors in clonidine’s effect is supported (Figures 3A–C). Accordingly, our data agree with the consensus that α_2A_-adrenoceptor is relevant to the spinal clonidine effect (Pertovaara, 2013).

In this regard, the effect of spinal clonidine on WDR cells was tested to give an electrophysiological correlate. At the spinal level, it is well-known (Urch and Dickenson, 2003) that these second-order neurons receive concomitant input from PAFs, and electrical stimulation of the peripheral RF produces a triphasic response corresponding to the activation of Aβ-, Aδ- and C-fibers (Figure 4), also a post-discharge can be elicited (Figure 4). This experimental approach let us analyze the impact of clonidine in nociceptive transmission, particularly how this ligand affects the firing of PAFs. As illustrated in the PSTHs of WDR cells (Figure 5) or the temporal course of neuronal activity (Figure 6), 10 nmol clonidine preferentially diminishes the neuronal firing associated with activation of nociceptive Aδ- and C-fibers, supporting the notion that spinal clonidine-induced inhibition via presynaptic action (i.e., on PAFs). In addition, the ongoing activity (i.e., the post-discharge) was also inhibited by 10 nmol clonidine. In direct support of these findings, *in vivo* (Sonohata et al., 2004) and *in vitro* (Pan et al., 2002; Kawasaki et al., 2003) electrophysiological recordings show that α_2_-adrenoceptor activation diminished the excitatory postsynaptic current evoked by PAF stimulation. At this point, we need to remember that post-discharge represents a late response of C-fibers to peripheral stimulation that can be endured by a traumatic injury leading to a neuronal hyperexcitability observed electrophysiologically as a wind-up (Woolf, 1996). However, under our experimental approach, we were unable to induce wind-up. Hence, an interesting question not addressed in the present work relates to how wind-up can be affected by noradrenergic transmission.

Accordingly, to the behavioral experiments, we also showed that presynaptic pharmacological blockade of α_2A_-adrenoceptors in WDR recordings is relevant to the effect of clonidine. Although these data agree with histological/molecular data showing the presence of α_2A_-adrenoceptors at the PAF level (i.e., Aδ- and C-fibers) (Stone et al., 1998; Birder and Perl, 1999), no direct *in vivo* evidence about a functional role of the presynaptic α_2A_-adrenoceptor subtype had been previously reported.

Regarding α_2B_-adrenoceptors, the behavioral experiments reject the involvement of this adrenoceptor subtype. As shown in Figures 3A–C supramaximal dose of imiloxan (α_2B_-adrenoceptor antagonist) does not affect clonidine’s antinociception. Current literature seems to support the notion that spinal α_2B_-adrenoceptor does not influence pain modulation (for refs., see Pertovaara, 2013; Llorca-Torralba et al., 2016; Bahari and Meftahi, 2019). For example, in an inflammatory pain model, Zhang et al. (2012) showed that spinal α_2B_-adrenoceptor blockade with imiloxan does not reverse the antinociception induced by enhancement of the descending noradrenergic tone, suggesting that this receptor subtype is not necessary for the noradrenergic analgesic effect. However, in neuropathic pain models involving ligature of spinal nerves (not tested in the present experiments) and using selective ligands, it seems that α_2B_-adrenoceptor acquire a role in the inhibition of nociception (Chu et al., 2015; Rodríguez-Palma et al., 2022) an effect probably related with cortical activation of this receptor subtype rather than spinal mechanism (Chu et al., 2015). In essence, our data suggest that spinal α_2B_-adrenoceptor does not play a role in tonic inflammatory and acute nociception.

In contrast, current data about the functional role of α_2C_-adrenoceptors inhibiting nociception is scarce, and as discussed by Pertovaara (2013), the impact of this receptor subtype in spinal nociception is unclear. These inconsistent data are partly due to the lack of selective compounds used to dissect the contribution of different α_2A/2B/2C_-adrenoceptor subtypes. For example, in transgenic mice, Fairbanks et al. (2002) showed that α_2C_-adrenoceptors have a subtle role in the moxonidine (a mixed I_1_ imidazoline/α_2C_-adrenoceptor agonist)-induced antinociception, whereas Malmberg et al. (2001) suggest that α_2A_- but not α_2C_-adrenoceptors are indispensable for the antinociceptive action of i.t., dexmedetomidine (a non-selective α_2A/2B/2C_-adrenoceptor antagonist).

In our behavioral experiments, we used JP 1302, a selective α_2C_-adrenoceptor antagonist (Sallinen et al., 2007; Proudman et al., 2022), to test the role of this receptor in clonidine-induced antinociception. According to Figures 3A–C i.t., JP 1302 was unable to block clonidine’s effect. Similar results were found in a neuropathic pain model (Rodríguez-Palma et al., 2022) or writhes-induced by sleep deprivation (Yaoita et al., 2018), showing that systemic JP 1302 does not preclude the behavioral antinociceptive action of α_2_-adrenoceptor agonists (ST-91 or tizanidine given systemically). However, when we analyzed the *per se* effects of the antagonist (Figures 3D–F), we found that in sharp contrast to BRL 44408 or imiloxan, i.t., JP 1302 diminishes the number of flinches induced by formalin (in phase I and II), implying that this antagonist has an antinociceptive effect during the formalin test.

### 4.3 The potential pronociceptive role of α_2C_-adrenoceptors in tonic nociception

In contradiction to the behavioral data, when we explored the effect of JP 1302 using an electrophysiological approach (Figures 7A–F), no effect was observed in the neuronal firing of WDR cells. This discrepancy could be attributable to the nature of the noxious stimulus used (i.e., tonic vs. acute) and may imply that JP 1302 exerts its antinociceptive action in a different way than clonidine not involving presynaptic or WDR cell inhibition. Admittedly, an interesting iteration to try to disentangle and give an electrophysiological correlate would have been to analyze the effect of these ligands on wind-up, taking into account that this neuronal process has been related with central sensitization due to recruitment of nociceptive circuits beyond of Aδ- and C-fibers activation. Regardless, if we consider that this compound exerts an antinociceptive effect *via* the blockade of α_2C_-adrenoceptors, it is interesting to note that α_2C_-adrenoceptors have been localized in non-noradrenergic brainstem descending fibers and postsynaptic sites in spinal interneurons (Stone et al., 1998; Olave and Maxwell, 2002). To our knowledge, no report about an antinociceptive *per se* action of selective α_2C_-adrenoceptor antagonists exist. Consequently, the question is: How can α_2C_-adrenoceptor blockade induce spinal antinociception?

Proudman et al. (2022) suggest that the selectivity of JP 1302 is reliable for dissecting between the α_2A/2B/2C_-adrenoceptor subtypes (see Table 1), but this antagonist also displays some affinity for α_1A_-adrenoceptors (p*K*_i_:6.2). Hence, we cannot ignore that interaction between JP 1302 and α_1A_-adrenoceptors could exist. Certainly, using an optogenetic approach, Kucharczyk et al. (2022) showed that activating a discrete noradrenergic descending pathway from locus coeruleus provokes a diminution of WDR activity, an effect blocked by prazosin (a non-selective α_1A/1B/1D_-adrenoceptor antagonist), suggesting a role for α_1_-adrenoceptors. However, in the formalin test, i.t., prazosin does not affect nocifensive behavior (Jeong et al., 2011; Park et al., 2011). Similarly, the clonidine-induced inhibition of WDR responses evoked by NMDA was unaffected by prazocin (Zhang et al., 1998). Indeed, subcutaneous formalin injection tends to decrease the [^3^H]-prazosin binding sites in the spinal cord (Nalepa et al., 2005), suggesting that under an inflammatory stimulus, the probability that this receptor could be exerting a pharmacological effect is minimal. Besides, using an acute (thermal) or neuropathic pain model (L_5_ - L_6_ nerve ligation), i.t., α_1_-adrenoceptor agonist (methoxamine) does not have any impact on the pain threshold (Nagasaka and Yaksh, 1990; Yaksh et al., 1995). Together these data support our contention that under our experimental conditions, the effect of JP-1302 may be mediated by its interaction with the α_2C_-adrenoceptor.

If spinal α_2C_-adrenoceptor blockade produces an antinociceptive effect in the formalin test but not in the electrophysiological experiments, the most straightforward interpretation of these findings may suggest (but does not prove) that under tonic nociception α_2C_-adrenoceptor activation counter-balance the antinociceptive effect of descending noradrenergic system. In this regard, subcutaneous formalin induces not only an increase in the endogenous descending noradrenergic activity (Ma et al., 2001; Sajadeianfard et al., 2005; Martins et al., 2013) but also a rise in the mRNA expression of α_2C_-adrenoceptor at spinal cord level (Yoon et al., 2011). Coupled with the evidence suggesting that this receptor is expressed in GABAergic interneurons (Holmberg et al., 1999; Olave and Maxwell, 2002), we hypothesize that during spinal tonic nociceptive input, activation of this receptor could, in turn, diminish the GABAergic transmission, favoring a pronociceptive state. This hypothesis gain weight considering that at the striatum level, it has been suggested that activation of α_2C_-, but not α_2A_-adrenoceptors inhibits GABA release (Holmberg et al., 1999; Zhang and Ordway, 2003). Hence, i.t., JP 1302 indirectly favors an antinociceptive effect by improving the activity of GABAergic neurons. To prove this hypothesis, and using the formalin test, we assessed the effect of spinal bicuculline (unspecific antagonist of the GABA_A_ receptors) on the JP 1302-induced antinociception.

At this point, we must emphasize that 0.3 nmol (0.11 μg) bicuculline *per se* does not influence flinching behavior induced by formalin (Figures 6F–H). This data agrees with previous reports showing that i.t., <0.3 μg bicuculline (<0.8 nmol) does not impact this nocifensive behavior (Dirig and Yaksh, 1995; Peng et al., 2015; Ryu et al., 2021). However, although some reports showed that spinal bicuculline is pronociceptive, this effect depends on the dose of bicuculline and the explored pain model. In the case of the formalin test, Kaneko and Hammond (1997) showed that at formalin concentrations <1%, an increase of flinches is induced by bicuculline, particularly at 0.3 μg (i.e., 0.8 nmol). Therefore, under our experimental conditions (1% formalin), the concentration used of bicuculline (0.3 nmol equivalents to 0.11 μg) seems to be adequate to evaluate the role of GABAergic participation in the JP 1302 effect.

The data show that the antinociception induced by JP 1302 was reversed with 0.3 nmol bicuculline (Figures 7H–J), suggesting that JP 1302 favors GABAergic transmission. *In vitro* evidence indicates that GABAergic neurons in the substantia gelatinosa can be hyperpolarized by noradrenaline (Hantman and Perl, 2005). Specifically, by recording GABAergic neurons at the superficial dorsal horn level using the patch-clamp technique, Gassner et al. (2009) proved that although the main effect of noradrenaline on GABAergic neurons is depolarization, a minor proportion of these cells are hyperpolarized *via* α_2_-adrenoceptors. In these studies, the specific receptor subtype was not identified. Together, these data support our contention that by inhibiting GABAergic neurotransmission, α_2C_-adrenoceptors may play a pronociceptive role (particularly during tonic nociception). The pronociceptive role of this receptor has also been suggested in *in vitro* assays by Chen et al. (2008), where they showed that α_2C_-adrenoceptor activation inhibits opioid release in the spinal cord; thus, blocking this receptor would induce antinociception.

### 4.4 A final consideration about the role of spinal α_2_-adrenoceptors subtypes in pain modulation

Our data suggest that during tonic pain, both α_2A_- and α_2C_-adrenoreceptors are activated, and one question that could arise is: exist a particular situation where α_2C_- surpass the antinociceptive effect of α_2A_-adrenoreceptors? Current evidence shows that the net impact of non-selective α_2_-adrenoceptor agonists is antinociception in acute and neuropathic pain conditions (for refs. see Bahari and Meftahi, 2019), pointing out the relevance of α_2A_-over α_2C_-adrenoreceptors. Certainly, we need to keep in mind that plastic changes in the spinal α_2_-adrenoreceptors occur under neuropathic pain and may explain, at least in part, the variability in the efficacy of α_2_-adrenoreceptor agonists depending on the stage of neuropathic pain (Yoon et al., 2011). At this point, it is worth mentioning that the expression of the different α_2A/2B/2C_-adrenoceptor subtypes at the spinal cord level under pathological pain has been analyzed. Stone et al. (1999) showed that after spinal nerve ligation, the immunostaining of α_2A_-adrenoceptor is reduced, whereas α_2C_-adrenoceptor is enhanced. Hayashida and Eisenach (2010) discussed the physiological relevance of this change, suggesting that under peripheral nerve injury, the function of α_2_-adrenoceptors can be altered. Furthermore, the relevance of α_2C_-adrenoreceptor in spinal nociception will be answered with a selective α_2C_-adrenoreceptor agonist (not available yet) or the use of more precise approaches (e.g., opto- and chemogenetics). Regardless, at the spinal level, the effect of clonidine in healthy and pathological pain is analgesia. Therefore, our results deserve further investigation.

Finally, at the spinal dorsal horn level, the role of noradrenergic transmission is far more complex than initially conceived. This complexity may depend on the type of adrenergic receptor stimulated, the localization of the receptor (e.g., PAFs, interneurons, second-order neurons), and the type of nociceptive input. For example, at the spinal trigeminal level, both α_2A_-, and α_2C_-adrenoceptors seem to inhibit nociceptive transmission (Villalón et al., 2012). More recently, it has been suggested that at the spinal cord level, the noradrenergic system elicits a fine-tunning of nociception by activation of α_1_-or α_2_-adrenoceptors; indeed, they proposed that spinal α_2_-adrenoceptors located presynaptically in the noradrenergic projections from the ventral LC can be modulating the firing of GABAergic interneurons (see Suppl Fig 2 in Kucharczyk et al. (2022)). Furthermore, as illustrated by Hickey et al. (2014), optogenetic experiments demonstrate that stimulation of different regions of locus coeruleus can evoke an antinociceptive or pronociceptive spinal effect; in this case, although i.t., atipamezole (a non-selective α_2A/2B/2C_-adrenoceptor antagonist) blocked the spinal clonidine-induced antinociception, this antagonist also seems to enhance the antinociceptive action of optogenetic stimulation of the noradrenergic system in the LC, but the mechanism/receptor by which atipamezole enhances antinociception was not further explored. This divergent effect of noradrenergic transmission on pain modulation has also been observed in healthy humans, where activation of α_2_-adrenoceptors (by yohimbine) elicits both antinociceptive and pronociceptive actions (Vo and Drummond, 2015).

### 4.5 Conclusion

Taken together, our data suggest that spinal α_2A_- and α_2C_-adrenoceptors exert a differential (opposite) effect on nociceptive transmission. Specifically, α_2A_-adrenoceptor activation on PAF inhibits the tonic and acute nociceptive input, whereas α_2C_-adrenoceptor activation appeared to inhibit GABAergic transmission, favoring nociception during a tonic (inflammatory) stimulus.

## Data Availability

The original contributions presented in the study are included in the article/supplementary material, further inquiries can be directed to the corresponding author.
